# Fetal magnetic resonance imaging features of syndromic megalencephaly: a systematic review of imaging-genotype associations

**DOI:** 10.1007/s00247-026-06747-3

**Published:** 2026-08-18

**Authors:** Stephanie Libzon, Moran Hausman-Kedem, Nilly Waiserberg, Liat Ben Sira

**Affiliations:** 1 Pediatric Neurology Institute, Dana-Dwek Children’s Hospital, Tel Aviv Sourasky University Medical Center (Ichilov), 6 Weizmann Street, Tel Aviv, 6423906 Israel; 2 Gray Faculty of Medical and Health Science, Tel Aviv University, Tel Aviv, Israel; 3 Department of Physical Therapy, School of Health Professions, Gray Faculty of Medical and Health Science, Tel Aviv University, Tel Aviv, Israel; 4 Division of Pediatric Radiology, Department of Radiology, Dana-Dwek Children’s Hospital, Tel Aviv Sourasky University Medical Center (Ichilov), 6 Weizmann Street, Tel Aviv, 6423906 Israel

**Keywords:** Fetus, Macrocephaly, Magnetic resonance imaging, Megalencephaly

## Abstract

Syndromic megalencephaly comprises a heterogeneous group of genetic disorders characterized by abnormal brain overgrowth and variable structural brain abnormalities. Prenatal recognition remains challenging because imaging findings overlap with those of isolated macrocephaly, while genotype–phenotype correlations are incompletely understood. This review summarizes the current literature on fetal magnetic resonance imaging (MRI) findings in molecularly confirmed syndromic megalencephaly and discusses their relationship with the underlying genetic pathways. The available evidence indicates that ventriculomegaly, malformations of cortical development, asymmetric lateral ventricles, hemimegalencephaly, and enlarged extra-axial spaces represent the most frequently reported prenatal MRI abnormalities, although their prevalence varies among genetic subgroups. Emerging imaging patterns suggest pathway-specific associations, particularly between the PIK3CA-AKT-mTOR pathway and hemimegalencephaly, and between RASopathies and enlarged extra-axial spaces. Current evidence is derived predominantly from case reports and small case series, underscoring the rarity of these disorders and the limited availability of systematic prenatal imaging data. Integrating fetal MRI findings with molecular diagnosis may improve prenatal diagnosis, facilitate targeted genetic testing, enhance prenatal counseling, and provide a foundation for future imaging-based diagnostic frameworks in syndromic megalencephaly.

**Graphical abstract:**

**Figure:**
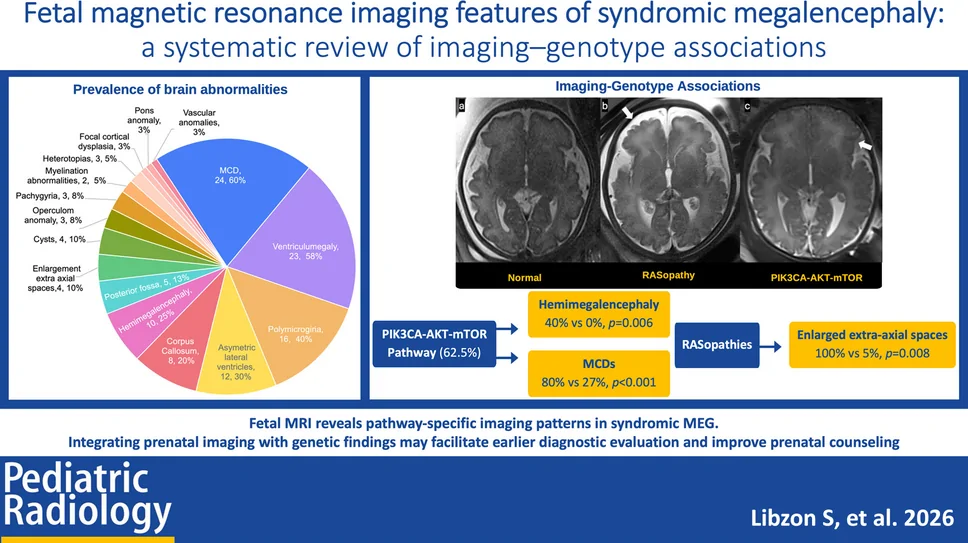

**Supplementary information:**

The online version contains supplementary material available at https://doi.org/10.1007/s00247-026-06747-3.

## Introduction

Megalencephaly is characterized by brain overgrowth due to an increased size and/or number of neurons and glia [1], with brain size exceeding two standard deviations above the age-related mean. Megalencephaly must be differentiated from other causes of macrocephaly, such as obstructive hydrocephalus, which cause an increase in head size without cerebral overgrowth.

**Fig. 1:**
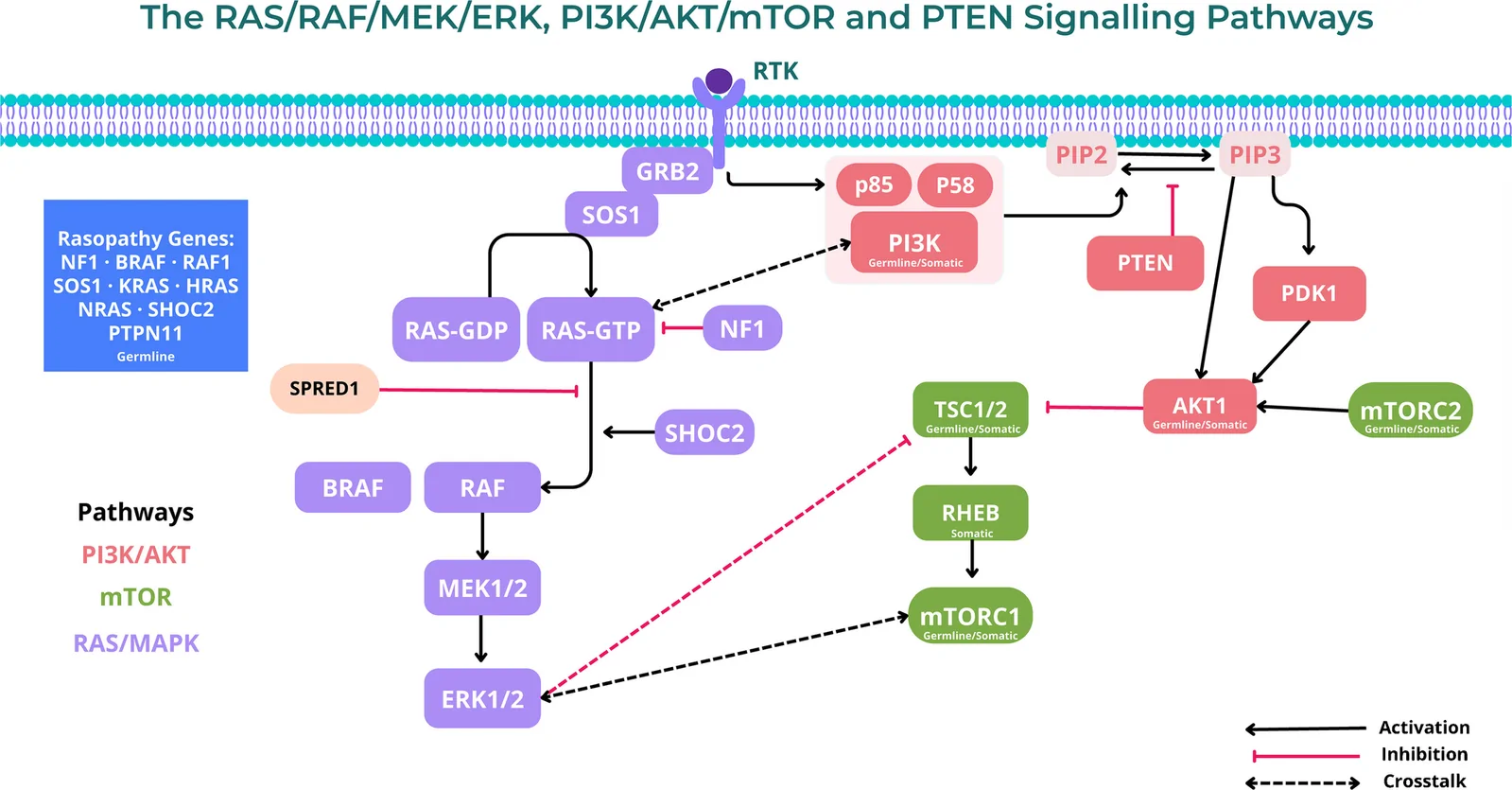
The RAS/RAF/MEK/ERK, PI3K/AKT/mTOR, and PTEN signaling pathways. Schematic representation of the major signaling pathways implicated in syndromic megalencephaly and developmental overgrowth disorders. Following activation of receptor tyrosine kinases (RTKs), the RAS/RAF/MEK/ERK pathway is initiated by the adaptor proteins GRB2 and SOS1, promoting the conversion of inactive RAS-GDP to active RAS-GTP and resulting in sequential activation of RAF, MEK1/2, and ERK1/2. In parallel, phosphatidylinositol 3-kinase (PI3K) converts phosphatidylinositol 4,5-bisphosphate (PIP2) to phosphatidylinositol 3,4,5-trisphosphate (PIP3), leading to activation of AKT and downstream mTOR signaling. PTEN acts as a negative regulator of this pathway by antagonizing PIP3 formation. Crosstalk between the MAPK and PI3K/AKT/mTOR pathways occurs through ERK-mediated modulation of TSC1/2, a key regulator of mTOR activity. AKT phosphorylates and inhibits TSC1/2, which normally suppresses mTOR activity by preventing RHEB activation. *Solid arrows* indicate activation, *flat-headed lines* indicate inhibition, and *dashed arrows* indicate pathway crosstalk

Megalencephaly may be non-syndromic or syndromic. Non-syndromic (benign familial) megalencephaly is typically associated with a favorable developmental outcome and often presents with mild macrocephaly (head circumference (*z*-score between +2 and +3 standard deviations (SD)) [2, 3]. More severe macrocephaly, particularly exceeding +3 SD, is more frequently associated with metabolic and degenerative diseases or with overgrowth syndromes [2, 3]. The latter is usually caused by gene disorders, mainly related to cellular growth regulation, including the *PI3K-AKT-mTOR* (PAM) pathway and *PTEN*-related disorders, the Ras/mitogen-activated protein kinase (MAPK) pathway (collectively referred to as RASopathies) (Fig. 1), the sonic-hedgehog (SHH) signaling pathway, and nuclear factor I (NFI)-related disorders [1–5]. Pathogenic or likely pathogenic (P/LP) variants in these pathways can be germline (present in gametes and all cells) or somatic, which are often undetectable by standard genetic testing. Syndromic megalencephaly may present with systemic or isolated neurologic manifestations, depending on the affected tissue.

Postnatal magnetic resonance imaging (MRI) in syndromic megalencephaly reveals characteristic features such as thick corpus callosum, cerebral asymmetry, polymicrogyria [6], focal cortical dysplasia [7], hemimegalencephaly [8], simplification of the cortical gyrification [9], white matter hyperintensities, ventriculomegaly [6], enlarged extra-axial spaces [9], dilated Virchow–Robin spaces, vascular malformations [7], and Chiari I malformation [6].

Antenatally, syndromic megalencephaly is often suspected based on extracerebral findings first seen on screening ultrasound, such as increased nuchal translucency and nuchal fold, cystic hygromas, and cardiac anomalies [2, 10] when present along with macrocephaly. While ultrasound remains the cornerstone of detection, macrocephaly is a common indication for fetal brain MRI. However, distinguishing benign causes from syndromic megalencephaly can be challenging, especially when imaging findings are inconclusive or nonspecific, as similar features may be present in other genetic syndromes. Whether postnatal head circumference thresholds can be directly extrapolated to the prenatal setting remains uncertain, as relatively mild fetal macrocephaly may reflect the early phase of progressive syndromic brain overgrowth, with head enlargement becoming more pronounced during late gestation and postnatal development. The prenatal characteristics of syndromic megalencephaly are poorly defined, with most insights derived from postnatal and neuro-sonographic studies. This is partly because macrocephaly often becomes apparent only in late pregnancy, in the third trimester, narrowing the window for timely genetic evaluation. This diagnostic gap limits early detection and counseling, particularly critical when pregnancy termination is being considered. This systematic review aims to assess the prevalence of brain abnormalities in fetuses with syndromic megalencephaly on brain MRI, and to relate them to underlying genetic pathways.

## Materials and methods

### Study design

**Fig. 2:**
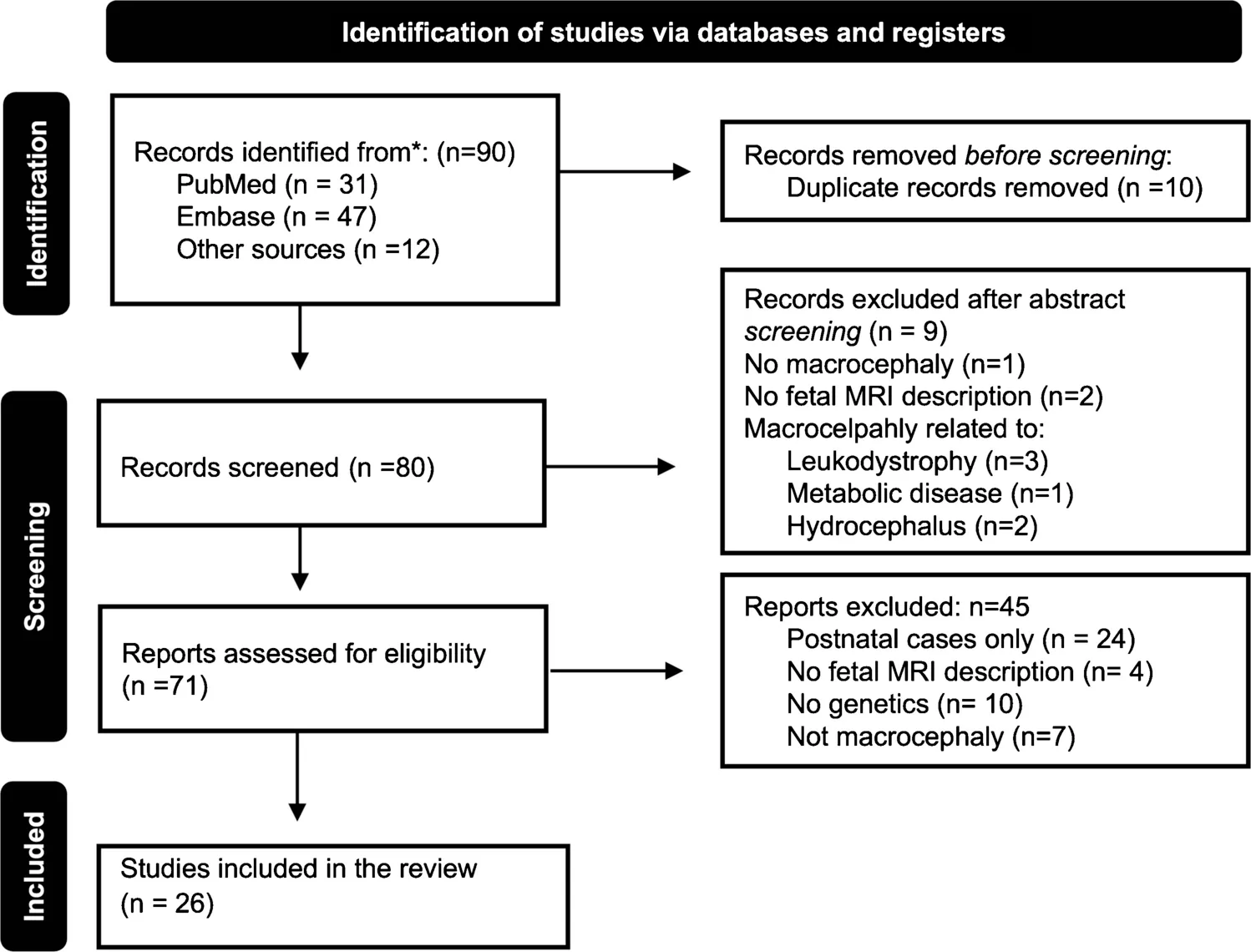
PRISMA flowchart

This systematic review was performed according to the Preferred Reporting Items for Systematic Reviews and Meta-Analyses (PRISMA) 2020 statement [11]. The protocol for this review was registered with the PROSPERO International Prospective Register of Systematic Reviews, bearing the registration number CRD420251041014. Two reviewers assessed studies for inclusion independently; disagreements were resolved through consensus. Study selection is detailed in the PRISMA flowchart (Fig. 2).

### Search strategy

We searched PubMed and Embase for articles published from January 2000 through April 2025 (search conducted April 30, 2025). Additional potentially relevant studies were identified through a manual search. The following search terms were used: (Megalencephaly OR Macrocephaly) AND (fetus disease OR prenatal) AND brain AND nuclear magnetic resonance AND (Genetic OR Overgrowth) NOT leukoencephalopathy OR leukodystrophy OR hydrocephalus OR metabolism. Searches were limited to English-language articles and human studies. The complete search strategy for each database is listed in Supplementary Material 1.

Definition of macrocephaly - for inclusion, macrocephaly was defined as a head circumference or FOD≥2SD above the GA mean, when such biometric data were available. In studies where biometric measurements were not reported, macrocephaly was based on descriptive terms such as “macrocephaly,” “megalencephaly,” or “increased head biometry.”

### Eligibility criteria

Eligible studies included those that addressed the following topics: (1) Fetuses; (2) Megalencephaly or Macrocephaly, absolute or relative, identified prenatally or postnatally; (3) Molecularly genetic diagnoses associated with syndromic megalencephaly, either during pregnancy or after birth; (4) Description and/or illustration of fetal brain MRI. The exclusion criteria included other reasons for macrocephaly not related to syndromic megalencephaly: (1) Fetuses with macrocephaly secondary to hydrocephalus and (2) Fetuses with macrocephaly due to metabolic disorders and degenerative disorders such as leukoencephalopathies and leukodystrophies. A complete list of excluded studies, along with the reasons, is provided in Supplementary Material 2.

### Study selection process

Two reviewers, S.L. (PhD student with expertise in fetal neuroradiology and 13 years of clinical and research experience) and N.W. (pediatric physical therapist and academic researcher with 30 years of experience), screened the initial search results independently, filtering articles based on their titles and abstracts against the eligibility criteria. Full-text articles of potentially eligible studies were independently assessed. Disagreements were resolved in discussion.

Given the rarity of syndromic megalencephaly with detailed prenatal imaging, we included all study types reporting original data, including case reports, case studies, and one narrative review. The narrative review was included because it provided original, extractable fetal MRI data not available elsewhere. Data from this review were treated as individual case-level data and evaluated using the same methodology and quality assessment methods as those used for the case reports and case series. Only those fetuses with confirmed syndromic megalencephaly, based on prenatal or postnatal genetic testing, accompanied by detailed fetal MRI findings, were included. Both liveborn fetuses and those with pregnancy termination were included. Potential duplicate cases were assessed by comparing available demographic, imaging, genetic, institutional, and publication characteristics across studies.

### Data extraction

Data were independently extracted by two reviewers, L.B.S. (pediatric neuroradiologist with 25 years of experience) and S.L. (PhD student with expertise in fetal neuroradiology with 13 years of clinical and research experience), using a standardized data extraction form. The following data were systematically extracted for every case: study characteristics; genetic investigation and findings, including the type of genetic test (chromosomal microarray and whole exome sequencing, targeted gene panel and targeted single-gene sequencing), the confirmed genetic diagnosis, and whether the identified variant was germline or somatic when reported; and maternal demographics. Detailed fetal MRI findings were recorded, including structural brain abnormalities such as (1) ventriculomegaly; (2) asymmetric lateral ventricles (irrespective of the presence of ventriculomegaly); (3) cysts: periventricular pseudo cysts, subependymal, interhemispheric, and Blake’s pouch cysts; (4) myelination abnormalities; anomalies of the (5) cavum septum pellucidum and (6) pons; (7) enlargement of the extra-axial spaces; (8) posterior fossa anomalies: cerebellar hemispheres and vermian atrophy or hypoplasia; (9) corpus callosum abnormalities: thinning of the corpus callosum and corpus callosum dysplasia; and (10) malformations of cortical development including heterotopias, hemimegalencephaly, focal cortical dysplasia, polymicrogyria, pachygyria, and abnormal operculization. For statistical analysis, individual malformations of cortical development were analyzed separately and also grouped into a composite variable, to increase the statistical power due to the small cohort size. This structured methodology allows for effective comparisons across studies, highlighting genetic-neuroimaging correlations in syndromic conditions.

### Risk of bias and quality assessment

Two authors (S.L. and N.W.) independently assessed study quality using the Joanna Briggs Institute (JBI) Critical Appraisal Checklists [12, 13], by Studies were classified as high quality (≥70%), moderate quality (50–69%), or low quality (<50%) (Supplementary Material 3). Disagreements were resolved through discussion and consensus.

### Statistical analysis

Descriptive statistics were calculated for all variables. Demographics, imaging findings, and clinical characteristics were summarized as counts and percentages for categorical variables, means±standard deviations (SD) for normally distributed continuous variables, and medians with interquartile ranges (IQR) for non-normally distributed variables. Categorical and continuous variables were compared using the *χ*^2^, Fisher’s exact test, Student’s *t*-test, and Mann–Whitney *U* test. Given the exploratory nature of this systematic review and the small sample size, we did not apply corrections for multiple comparisons. All statistical tests were two-tailed; *P*<0.05 was considered statistically significant. Statistical analyses were conducted using SPSS v28 (IBM Corp., Armonk, NY).

## Results

### Study selection

The systematic search of PubMed and Embase identified 90 records. After removal of duplicate entries, 80 articles remained for title and abstract screening. Of these, nine articles were excluded due to absence of fetal MRI description (*n*=2), lack of macrocephaly (*n*=1), or macrocephaly related to other conditions (leukodystrophies, *n*=3; hydrocephalus, *n*=3; metabolic disorders, *n*=1). A total of 71 articles, therefore, met the predetermined eligibility criteria and were included for further evaluation. Full-text review confirmed the inclusion of 26 articles reporting 40 fetuses that met the predefined inclusion criteria. The study selection process and reasons for exclusion are summarized in the PRISMA flow diagram (Fig. 2).

### Study design and characteristics of the studies

All 26 articles that met the inclusion criteria were published from 2010 onward. Seventeen manuscripts (65.5%) were case reports, seven (26.9%) were case series, one (3.8%) was a cohort study, and one (3.8%) was a narrative review (Table 1).

**Table 1:** Genetic and neuroimaging characteristics of fetuses with syndromic megalencephaly, presented by genetic pathway and year of publication (*n* = 40)

Case #	Study	Publication Type (total number of cases)	Included cases	Original case # Original case #	Referral Indication for Fetal MRI	Weeks gestational age at MRI	Fetal MRI Findings	Sex	Chromosomal microarray	exome sequencing type: pathogenic/likely pathogenic variant; germline/somatic^c^	Other genetic testing	Genetic pathway	Prenatal head circumference ^a^ +/- megalencephaly^b^	Postnatal head circumference ^a^ +/- megalencephaly^b^
1	Andrade (2024) [22]	Case report (*n*=1)	1/1	1	Asymmetry of the Lateral Ventricles	24, 32	Asymmetry of the lateral ventricles	M	NS	Single: *PIK3CA,* c.2176G>A; p.Glu726Lys		PIK3CA-AKT-mTOR	>2SD	>2SD
2	Giovannangeli (2024) [14]	Case report (*n*=1)	1/1	1	Macrosomia, Unilateral VM, abnormal gyration	27	Absence anterior CC & thick posterior segment	M	﻿Negative	Trio*:AKT1,* c.49G>A; p.Glu17Lys de novo; germline		PIK3CA-AKT-mTOR	Megalencephaly	NS
3	Papaioannou and Garel (2023) [23]	Narrative Review (*n*=12)	1/12	Fig. 2	Macrocephaly	31	Polymicrogyria, Frontal bossing	F	NS	Missense variant in the *PIK3R2*		PIK3CA-AKT-mTOR	>2SD	NS
4	Porwal (2022) [24]	Case report (*n*=1)	1/1	1	Unilateral VM, midline shift	26	VM (16mm), asymmetry of the lateral ventricles, hemimegalencephaly	F	NS	NS	Test not specified: *PIK3CA;* somatic	PIK3CA-AKT-mTOR	>2SD	+2.7SD
5	Mori (2022) [25]	Case report (*n*=1)	1/1	1	Polyhydramnios, macrocephaly, malformation of cortical development	27	Focal cortical dysplasia	M	NS	Trio: *AKT3,* c.233A>G; p.Gln78Arg, heterozygous, de novo; germline		PIK3CA-AKT-mTOR	Macrocephaly	+8.19SD
6	Di Stasi (2021) [17]	Case series (*n*=3)	1/3	3	Susp hemimegalencephaly	21	VM, asymmetry of the lateral ventricles, cerebellar hemisphere, hemimegalencephaly	NS	NS	Single: *PIK3CA*, c.1633G>A; p.Glu545Lys; somatic		PIK3CA-AKT-mTOR	Increased biometry on MRI	NA
7	Goergen (2021) [26]	Case series (*n*=22)	2/22	2	NS	31+3	Operculum anomaly, polymicrogyria	NS	NS	NS	Test not specified: *PIK3CA,* c. 2176G>A, pathogenic	PIK3CA-AKT-mTOR	+6SD (FOD)	NS
8				5	NS	25	Operculum anomaly, polymicrogyria	NS	NS	NS	Test not specified: *MTOR,* c.5930_5931del A>G; p.Thr1977Lys. De novo	PIK3CA-AKT-mTOR	+6SD (FOD)	NS
9	Bourgon (2021) [15]	Case series (*n*=21)	13/21	1	﻿Macrocephaly and/or limb Overgrowth with/without VM	32	Mild VM, polymicrogyria	F	NS	*PIK3R2*, c.1117G>A; p.Gly373Arg		PIK3CA-AKT-mTOR	+5.8 SD + megalencephaly	+3.33SD + megalencephaly (PM)
10				3		NS	Mild VM, CC, polymicrogyria	F	NS	*PIK3R2*, c.1117G>A; p.Gly373Arg; somatic		PIK3CA-AKT-mTOR	+3.6 SD + megalencephaly	+2.7SD + megalencephaly (PM)
11				4		NS	Severe VM, Posterior fossa, CC, polymicrogyria	F	NS	*MTOR*, c.7255G>A; p.Glu2419Lys		PIK3CA-AKT-mTOR	+4.1 SD + megalencephaly	3.8SD + megalencephaly (PM)
12				5		NS	Mild VM, polymicrogyria	M	NS	*AKT3*, c.963T>G; p.Asn321Lys; germline		PIK3CA-AKT-mTOR	+4.2 SD + megalencephaly	+4.8SD + megalencephaly (PM)
13				6		34	CC, polymicrogyria	F	NS	*PIK3R2*, c.1117G>A; p.Gly373Arg		PIK3CA-AKT-mTOR	+5.5 SD + megalencephaly	+2.5SD + megalencephaly (PM)
14				7		NS	NA	F	NS	*PIK3CA*, c.2727C>A; p.Phe909Leu		PIK3CA-AKT-mTOR	+4.7 SD + megalencephaly	+2.4SD - megalencephaly (PM)
15				8		NS	VM, asymmetry of the lateral ventricles, Posterior fossa, hemimegalencephaly, polymicrogyria	F	NS	*PIK3CA*, c.3140A>G; p.His1047Arg		PIK3CA-AKT-mTOR	+3.5SD - megalencephaly	+2.3SD - megalencephaly (PM)
16				9		23	VM, asymmetry of the lateral ventricles, hemimegalencephaly	F	NS	*PTEN*, c.139A>G; p.Arg47Gly; germline		PIK3CA-AKT-mTOR	+2.5 SD - megalencephaly	NA
17				10		NS	VM, asymmetry of the lateral ventricles, hemimegalencephaly	M	NS	*PIK3CA*, c.1633G>A; p.Glu545Lys		PIK3CA-AKT-mTOR	+3.6 SD - megalencephaly	+5SD - megalencephaly (PM)
18				12		NS	VM, asymmetry of the lateral ventricles, Posterior fossa, hemimegalencephaly, polymicrogyria	F	NS	*PIK3CA*, c.271G>A; p.Asp91Asn		PIK3CA-AKT-mTOR	+11.3 SD - megalencephaly	+7.9SD - megalencephaly (PM)
19				14		NS	VM, asymmetry of the lateral ventricles, hemimegalencephaly, polymicrogyria	M	NS	*PIK3CA*, c.1633G>A; p.Glu545Lys; somatic		PIK3CA-AKT-mTOR	+2.26 SD - megalencephaly	+2.5SD - megalencephaly (PM)
20				15		NS	VM, asymmetry of the lateral ventricles	M	NS	*PIK3CA*, c.1633G>A; p.Glu545Lys		PIK3CA-AKT-mTOR	+8.2 SD + megalencephaly	+8.3SD - megalencephaly (PM)
21				16		NS	VM, asymmetry of the lateral ventricles, hemimegalencephaly	F	NS	*PIK3CA*, c.328_330del p.Glu110del; somatic		PIK3CA-AKT-mTOR	+3.5 SD -megalencephaly	+4.2SD - megalencephaly (PM)
22	Abell (2020) [27]	Case report (*n*=1)	1/1	1	﻿Enlarged 3^rd^ ventricle, macrocrania, ambiguous genitalia	27	VM, dysmorphic lateral ventricles, heterotopia, polymicrogyria	F	Negative	Single ES: *AKT1,* c.49G>A; p.Glu17Ly; germline		PIK3CA-AKT-mTOR	megalencephaly	>2SD
23	Quinlan-Jones (2017) [28]	Case report (*n*=1)	1/1	1	Macrocephaly, asymmetric cerebral hemispheres	23	Hemimegalencephaly	M	Negative	NS	*Targeted next generation sequencing (panel):PIK3CA,* c.1633G>A; p.Glu545Lys; germline	PIK3CA-AKT-mTOR	>2SD	>2SD
24	Swarr (2013) [29]	Case report (*n*=1)	1/1	1	Hydrops, asymmetry of the lateral ventricles, macrocephaly	23+6	Asymmetry of the lateral ventricles	M	NS	NS	Targeted single-gene sequencing: heterozygous *PIK3CA,* c.501G>C; p.R115P; from skin biopsy	PIK3CA-AKT-mTOR	1.28-1.65SD	2SD
25	Yu (2025) [30]	Case series (*n*=14)	1/14	3	Macrocephaly and ventriculumegaly	30+3	Mild VM, gray matter heterotopias	M	Negative	Trio:*PTEN, c.389G > A; p.Arg130Gln*		PIK3CA-AKT-mTOR	+3.27	Macrocephaly
26	Beltrami (2022) [31]	Case report (*n*=1)	1/1	1	Elevated NT, polyhydramnios, macrocephaly	NS	Asymmetry of the lateral ventricles, Enlargement of EA spaces	F	Negative	*Trio: NRAS, missense c.34G>A; p.Gly12Ser; germline*		RASopathies	>2SD	>2SD
27	Hague (2017) [32]	Case report (*n*=1)	1/1	1	Polyhydramnios, macrocephaly	NS	VM (14 mm), Enlargement of EA spaces	M	NS	NS	Targeted single-gene sequencing: *HRAS,* p.Gly12Ser; germline	RASopathies	+2SD	+2.7SD
28	Chen (2014) [33]	Case report (*n*=1)	1/1	1	Polyhydramnios, macrocephaly, VM	31	VM (13/15mm)	F	Microdeletion in 5q35.2-q35.3 (*NSD1*)	NS		Gene transcription	1.96-2SD	Macrocephaly
29	Speksnijder (2013) [16]	Case report (*n*=1)	1/1	1	VM, tear drop shape lateral ventricles, agenesis of the CC, syndactyly	22	CC, cysts	F	Negative	Trio ES: *GLI3,* c.2786T>C; p.leu929Pro, missense, de novo; germline		SHH	+4.5SD	>2SD
30	Shear (2024) [34]	Case report (*n*=2)	1/2	1	Polyhydramnios, SVT	32	Moderate VM	M	Negative	Negative	Repeat-primed polymerase chain reaction: Heterozygous expanded allele >200 repeats- *DM1*	Other genetic syndromes	NS	Macrocephaly
31	Libzon (2024) [35]	Cohort Study (*n*=60)	2/60	16	VM	NS	Severe VM, thin CC, polymicrogyria		Negative	Trio: *NFIA* c.256G>A; germline		Other genetic syndromes	+1.9SD	NS
32				40	Macrocephaly, polyhydramnios	NS	Mild VM		Negative	*CAMTA1*, c.45+1G>T		Other genetic syndromes	Macrocephaly	NS
33	Lei (2023) [36]	Case report (*n*=2)	1/2	2	VM	>24+5	Severe VM	NS	Negative	Trio: Heterozygous *PPP2R1A*, c.547C > T (p. Arg183Trp), de novo; germline		Other genetic syndromes	>2SD	NS
34	Liu (2023) [37]	Case report	1/1	1	Macrocephaly	28	Pachygyria	M	Negative	Trio: *TAOK1,* c.806G>A; p.Arg269Gln; germline		Other genetic syndromes	+3.4SD	Large head circumference (PM)
35	Neri (2022) [38]	Case series (*n*=6)	1/6	1	Choroid plexus cysts, prominent lateral ventricles, posterior fossa cyst	20	Cysts	F	NS	*WASHC5*, c.232C>T; c.2489G>A , heterozygous; germline		Other genetic syndromes	NS	Macrocephaly
36	Malinger (2021) [39]	Case report (*n*=1)	1/1	1	Mild polyhydramnios, borderline macrocephaly	30	NA	M	NS	Trio: *CDH3* missense heterozygous, c.5111C>T; p.Ser1704Leu de novo; germline		Other genetic syndromes	+3.2SD	NS
37	Del Campos Braojos and Guimaraes (2018) [40]	Case report (*n*=2)	1/2	1	Macrocephaly, susp CC agenesis	21	Dysplastic CC, abn. Lamination, GE, polymicrogyria, myelination abn.	NS	NS	*KMT2D*, c.9782A>G; p.Q3261R; germline		Other genetic syndromes	>2SD	NS
38	Saliba (2018) [18]	Case series (*n*=4)	1/4	2	VM	27	VM, cerebellar vermis dysgenesis and cerebellar hypoplasia, brainstem	M	NS	NS	Targeted s*ingle-gene* sequencing*: FGFR2* heterozygous, c.1052>G, p.Ser351Cys, de novo	Other genetic syndromes	NS	Macrocephaly (PM)
39	Rodan (2018) [41]	Case series (*n*=4)	1/4	4	Polyhydramnios, macrocephaly	34+1	VM, Enlargement of EA spaces, myelination anomalies, cysts, polymicrogyria	M	Negative	Trio: *ODC1,* c.1242-1263del22; p.Trp414*, de novo; germline		Other genetic syndromes	+2SD	NA
40	Valduga (2015) [42]	Case report (*n*=2)	1/2	II-5	High rhythmic fetal movements	38	Enlargement of EA spaces, CC, cysts, operculum, polymicrogyria	M	Homozygous deletion in 16q23.1 (*WWOX*)	NS		Other genetic syndromes	Macrocephaly	+2.5SD

Only the first author is given for each study

^a^Head circumference is presented in standard deviations (SD). When originally reported as a percentile, values were converted to SD; the 97th percentile was considered equivalent to +2SD. When head circumference was not provided numerically, a descriptive term from the original article is included

^b^With or without the presence of megalencephaly, if described

^c^Somatic variant was defined when described as somatic, or when the variant was not found in the amniocytes culture

*abn*, abnormal, *CC* corpus callosum, *EA* extra-axial spaces, *GE* ganglionic eminence, *LP* likely pathogenic, *MCAP* Megalencephaly-Capillary Malformation syndrome, *NS* not specified, *PM* postmortem, *SD* standard deviation, *SHH* Sonic Hedgehog, *VM* ventriculomegaly

### Risk of bias assessment and quality assessment

According to the JBI Prevalence Critical Appraisal Tool, 20 studies (76.9%) were of high quality, and 6 (23.1%) were of moderate quality. The specific characteristics of the included studies are detailed in the supplements (Supplementary Material 3).

### Demographic and clinical data

Among the 40 fetuses analyzed, half were male 17/33, 51.5%. Maternal age at pregnancy was reported for 16 cases, with a median age of 32.5 years (IQR 28.25, 36.5). Fifteen fetuses (37.5%) were born, one died in utero (2.5%), and 24 (60%) pregnancies were terminated. Macrocephaly was identified prenatally in most cases (37/40, 92.5%). Among the three fetuses lacking prenatal head circumference data, postnatal assessments confirmed macrocephaly (Table 1, cases 30, 35, and 38).

Molecular diagnoses were established using exome sequencing in 28 cases (70%), targeted single-gene sequencing in 3 (7.5%), chromosomal microarray in 2 (5%), targeted gene panel in 1 (2.5%), and repeat-primed polymerase chain reaction in 1 (2.5%). The genetic testing modality was not specified in the remaining five cases (12%). Pathogenic/likely pathogenic variants were identified in nine genes across four major genetic pathways associated with macrocephaly: variants in the PAM pathway (25/40,62.5%); *PIK3CA* (*n*=13), *PIK3R2* (*n*=4), *AKT* (*n*=4), *MTOR* (*n*=2), and *PTEN* (*n*=2), variants related to RASopathies were found in two cases (2/40, 5%): *NRAS* (*n*=1) and *HRAS* (*n*=1), and SHH signaling pathway: *GLI3* (*n*=1), and a microdeletion of 5q35.2-q35.3 including *NSD1* gene associated with Sotos syndrome was identified in one case each (2.5%). The remaining 11 cases (11/40, 27.5%) involved P/LP variants in the following genes related to other genetic syndromes: *DM1*,* PPP2R1A*,* TAOK1*,* WASHC5*,* CDH3*,* CAMTA1*,* NFIA*,* KMT2D*, and* ODC1* (Table 1).

### Fetal magnetic resonance imaging findings

All studies included in this review utilized conventional fetal MRI sequences to assess their participants. The gestational age (GA) at the time of prenatal MRI was provided in 26 cases and was performed at a median of 27 weeks gestational age (IQR, 23.75, 31.25 weeks).

The most common brain abnormality was ventriculomegaly (23/40, 57.5%). The extent of ventricular dilatation was reported in 12/23 (52.2%) cases: mild (<12 mm, 5/12, 41.7%), moderate (12–15 mm, 3/12, 25%), and severe (>15 mm, 4/12, 33.3%). In the remaining 11 cases, the severity of ventriculomegaly was not specified. Ventriculomegaly was found as an isolated finding, accompanied by macrocephaly, in 17.4% of cases (4/26).

**Fig. 3:**
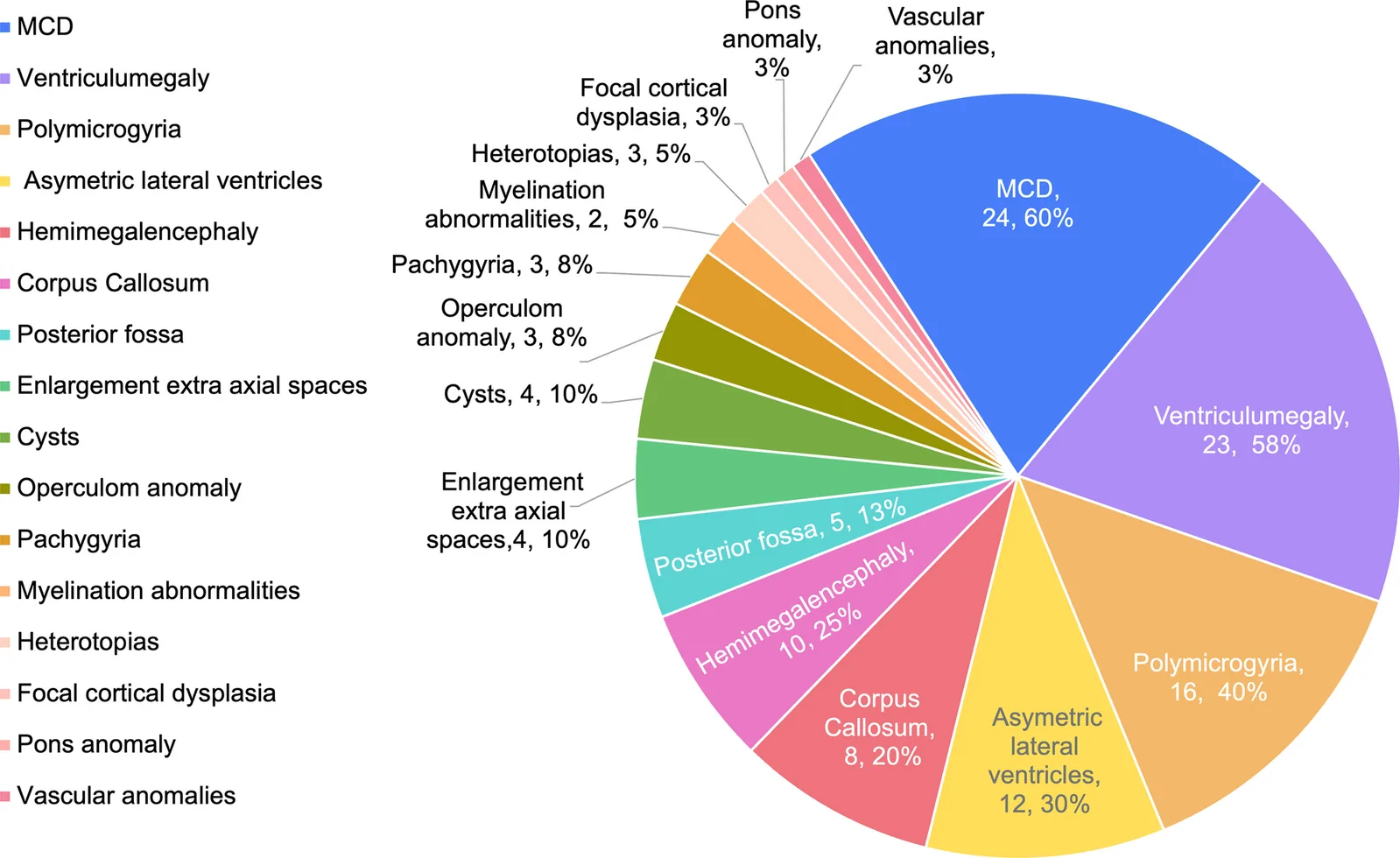
“Pie chart” illustrating the prevalence of brain abnormalities identified on fetal magnetic resonance imaging in 40 fetuses with syndromic megalencephaly

Polymicrogyria occurred in 16 cases (40%), followed by asymmetric lateral ventricles (*n*=12, 30%), hemimegalencephaly (*n*=10, 25%), corpus callosum (*n*=8, 20%) and posterior fossa anomalies (*n*=5, 12.5%), enlarged extra-axial spaces and cysts in 4 each (10%), and anomalies of the operculum (*n*=3, 7.5%). Pachygyria, heterotopias, and myelination abnormalities in 2 cases each (4.8%), while focal cortical dysplasia, vascular malformation, and anomalies of the pons were seen in 1 case each (2.5%). As a group, malformations of cortical development were present in 24 cases (60%) (Fig. 3).

### Genotype-phenotype associations

Eight fetuses (20%) had anomalies of the corpus callosum, four of which were linked to the PAM pathway. One fetus with Proteus syndrome, due to a P/LP variant in *AKT1*, had an absence of the anterior corpus callosum and a thickened posterior segment [14]. In three cases, the nature of the corpus callosum abnormality was not specifically characterized [15]. One case with suspected agenesis of the corpus callosum and an interhemispheric cyst, confirmed postnatally, was found to have a P/LP variant in *GLI3* [16]. The remaining cases included two fetuses with a thin corpus callosum associated with P/LP variants in *NFIA* and *WWOX*, and one fetus with a dysplastic corpus callosum linked to a P/LP variant in *KMT2D*.

Five fetuses presented with posterior fossa anomalies. Of these, 4/5 (80%) were linked to PAM pathway P/LP variants. One fetus with a P/LP variant in *PIK3CA* had hemimegalencephaly and an enlarged and ipsilateral dysplastic cerebellar hemisphere [17]. The other three cases had no description of the exact anomaly [15]. One fetus with a P/LP variant in *FGFR2* presented with cerebellar vermis dysgenesis and cerebellar hypoplasia [18].

**Fig. 4:**
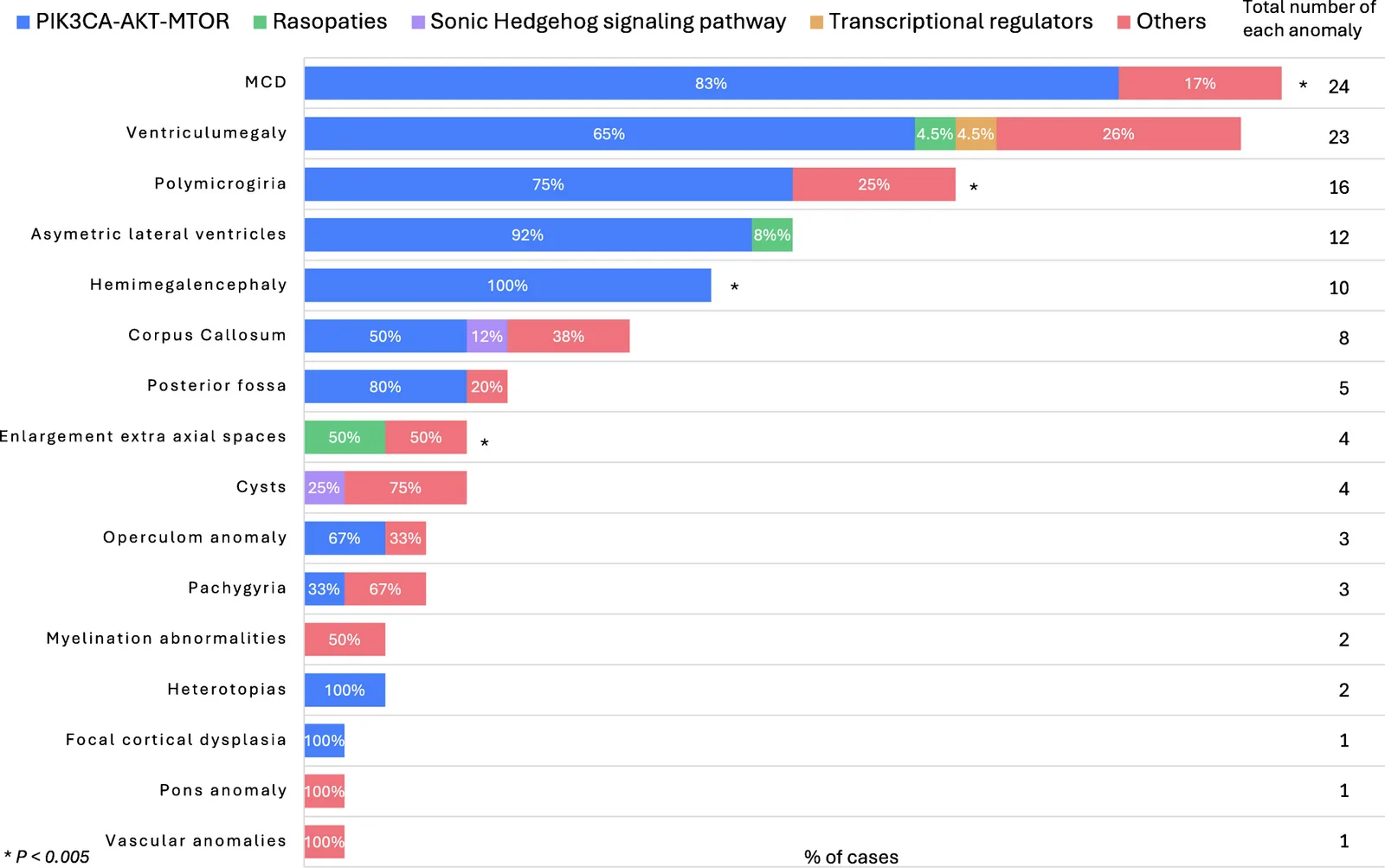
Distribution of fetal brain anomalies by genetic pathway in 40 fetuses with syndromic megalencephaly. Each *horizontal bar* represents the percentage of cases within a specific anomaly attributed to a given genetic pathway. The total number of fetuses with each anomaly is listed to the right of each bar. Statistically significant associations (*P*<0.05) are marked with an *asterisk* (*)

**Fig. 5:**
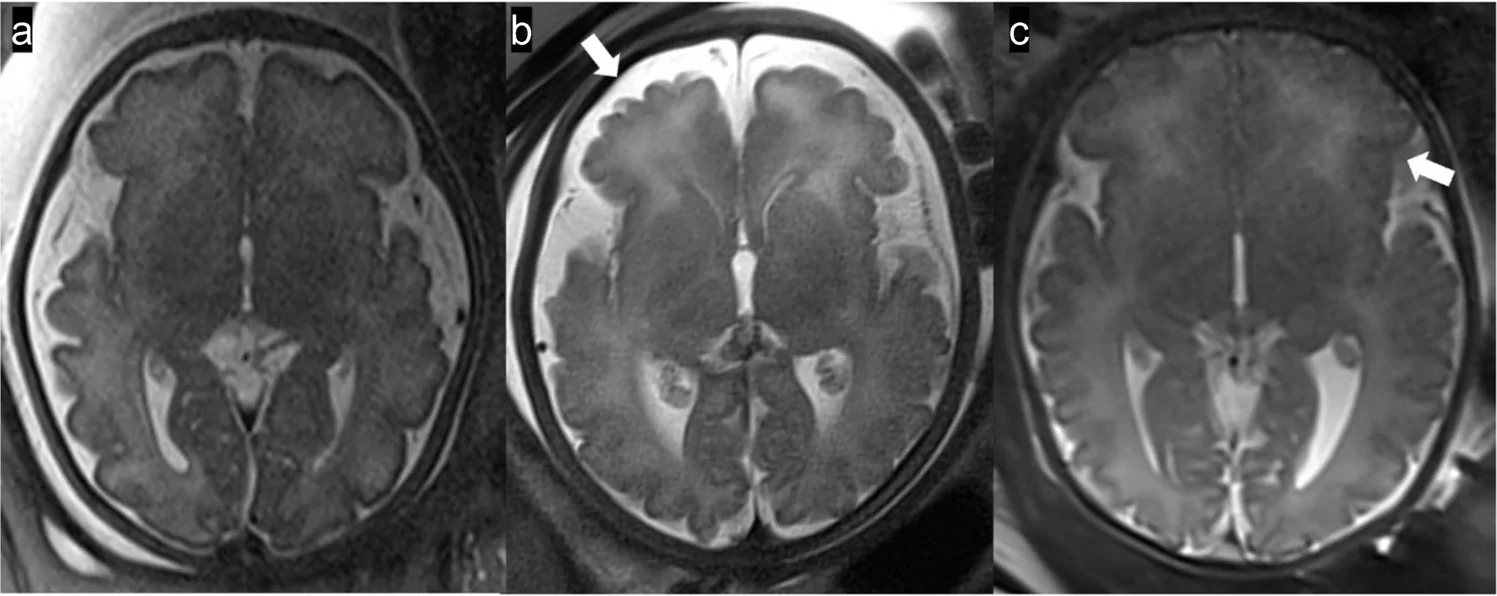
Illustrative cases of the main results of the systematic review. Magnetic resonance imaging (MRI) of three cases evaluated at Tel Aviv Medical Center, demonstrating the main results of the systematic review, in axial T2-weighted imaging: (**a**) MRI at 34+1 weeks gestational age of a male fetus with supratentorial biometry of +2SD, with normal whole exome sequencing, showing normal brain MRI, with enlargement of the extra-axial spaces in the peri-opercular region. **b** MRI at 35+3 weeks gestational age of a male fetus with supratentorial biometry of +3.7SD, diagnosed with a P/LP variant in *RRAS2* (Noonan syndrome), showing enlargement of the extra-axial spaces most prominent in the interhemispheric, frontal, and peri-opercular regions. Additionally, irregular small frontal gyri, suspected with dysgyria/polymicrogyria (*arrow*). **c** MRI at 34+6 weeks gestational age of a male fetus with supratentorial biometry of +3SD, diagnosed with a P/LP variant in *PIK3R2* showing abnormal operculum, especially on the left, with small thick frontal gyri consistent with polymicrogyria (*arrow*)

Hemimegalencephaly was identified exclusively in fetuses with P/LP variants in PAM patway (*P*=0.006). Malformations of cortical development were significantly associated with this pathway, occurring in 80% of cases compared to 26.7% in other genetic syndromes (*P*<0.001). Polymicrogyria was more common in this group, observed in 48% (12/25) of cases, compared to 26.7% (4/15) in fetuses with other genetic conditions, although the difference was not statistically significant. Asymmetric lateral ventricles were noted in 44% (11/25) of PAM cases and 50% (1/2) of RASopathy cases (*P*=0.022). Enlargement of extra-axial spaces was found in all cases with a RASopathy (2/2, 100%) and in two cases with other genetic syndromes due to a P/LP variant in *ODC1* and *WWOX* (2/38, 5.3%), but was not observed in any other genetic pathways (*P*=0.008). The incidence of brain abnormalities across different genetic pathways is presented in Table 2 and Fig. 4. Three illustrative cases presenting the main results are shown in Fig. 5.

**Table 2:** Prevalence of brain anomalies in syndromic megalencephaly associated with genetic pathway in 40 fetuses in the literature review

Pathway	Imaging Patterns	*n* (%)	Mutated gene	Imaging Patterns	*n* (%)
PIK3CA-AKT-mTOR (PAM) (*n*=25)	Malformations of cortical development	20 (80%)	***PIK3CA*** **(*n*=12)**	Asymmetric lateral ventricles	9 (75%)
				Malformations of cortical development	8 (67%)
	Ventriculomegaly	15 (60%)		Ventriculomegaly, hemimegalencephaly,	7 (58%)
				Polymicrogyria	4 (33%)
	Polymicrogyria	12 (48%)		Posterior fossa	3 (25%)
				Anomalies of the operculum	1 (8.3%)
	Asymmetric lateral ventricles	11 (44%)	***PIK3R2*** **(*n*=4)**	Malformations of cortical development, polymicrogyria	4 (100%)
				CC, Ventriculomegaly	2 (50%)
	Hemimegalencephaly	10 (40%)	***AKT*** **(*n*=4)**	Malformations of cortical development	3 (75%)
				Ventriculomegaly, polymicrogyria	2 (50%)
	CC, posterior fossa	4 (16%)		CC, heterotopia, pachygyria, focal cortical dysplasia	1 (25%)
			***MTOR*** **(*n*=3)**	Malformations of cortical development	3 (100%)
	Anomalies of the operculum, heterotopia	2 (8%)		polymicrogyria, ventriculomegaly, hemimegalencephaly	2 (67%)
				Anomalies of the operculum, asymmetric lateral ventricles, CC, posterior fossa anomaly	1 (33%)
	Focal cortical dysplasia, Pachygyria	1 (4%)	***PTEN*** **(*n*=2)**	Malformations of cortical development, ventriculomegaly	2 (100%)
				Asymmetric lateral ventricles, hemimegalencephaly, heterotopia	1 (50%)
RASopathies (*n*=2)	Enlargement EA spaces	2 (100%)			
	Ventriculomegaly, asymmetric lateral ventricles	1 (50%)			
Gene transcription (Sotos) (*n*=1)	Ventriculomegaly	1 (100%)			
Sonic Hedgehog (SHH) (*n*=1)	Ventriculomegaly, CC, cysts	1 (100%)			
Other genetic syndromes (*n*=11)	Ventriculomegaly	6 (54.5%)			
	Malformations of cortical development	4 (36%)			
	Polymicrogyria	4 (36.4%)			
	CC, cysts	3 (27.3%)			
	Myelination abn., enlargement EA spaces, pachygyria	2 (18.2%)			
	Anomalies of the operculum, posterior fossa, pons	1 (9.1%)			

*CC* corpus callosum, *EA* extra-axial, *SHH* Sonic Hedgehog

## Discussion

This systematic review analyzed MRI findings in 40 fetuses with molecularly confirmed syndromic megalencephaly, reported across 26 publications. The most frequently reported neuroimaging findings were ventriculomegaly (57.5%), mostly mild, polymicrogyria (40%), and asymmetric lateral ventricles (30%). While many findings were nonspecific and overlapped across genetic etiologies, distinct imaging patterns emerged that correlated with underlying molecular pathways. Malformations of cortical development were associated with the PAM pathway; hemimegalencephaly was exclusively observed in this group, occurring in 40% of cases. Enlarged extra-axial spaces were notably associated with RASopathies.

Ventriculomegaly and asymmetric lateral ventricles are frequently observed findings and are not specific to any single genetic cause; they overlap across multiple syndromic pathways and are also prevalent in typically developing fetuses. In this study, ventriculomegaly was diagnosed in 23/40 (57.5%) of the cases. In four cases, it was the sole radiological finding, in addition to macrocephaly. From these four, three had a genetic syndrome (*PPP2R1A*,* DM1*,* CAMTA1*), and one was diagnosed with Sotos syndrome due to a P/LP variant in *NSD1*. Asymmetric ventricles occurred in 12/40 (30%) and were “isolated” in addition to macrocephaly, in only 2 cases with a P/LP variant in *PIK3CA*. This underlines the importance of recognizing that the combination of macrocephaly with ventriculomegaly or asymmetric lateral ventricles may suggest an underlying genetic syndrome, with subtle MRI findings that are not well defined, and should not be dismissed as benign or incidental, warranting genetic evaluation and longitudinal imaging follow-up. These findings highlight the complexity of prenatal MRI in syndromic megalencephaly, emphasizing the need for thorough radiologic and genetic assessments conducted in a multidisciplinary manner for this unique population. Therefore, a holistic, multidisciplinary approach to fetal imaging should be adopted, including detailed assessments throughout the pregnancy, a genetic context to enhance diagnostic precision, and optimizing prenatal counseling.

In this cohort, P/LP variants in the PAM pathway were the most frequent genetic cause of syndromic megalencephaly, with *PIK3CA* the most affected gene. Hemimegalencephaly was observed only in cases in the PAM pathway. Furthermore, an association was found between this pathway and asymmetric lateral ventricles and malformations of cortical development, occurring in approximately half of fetuses with a P/LP variant in *PIK3CA*. These prenatal findings are consistent with the postnatal imaging features described in children with *PIK3CA*-related overgrowth spectrum (PROS) [19, 20], suggesting that the full spectrum of brain malformations is often detectable before birth.

The timing of fetal MRI acquisition is crucial for detecting cortical malformations. Polymicrogyria typically emerges during late neuronal migration and may not be evident before 24 weeks of gestational age. Additionally, polymicrogyria may present subtly as the absence of normal sulcation, delayed sulcation, or as abnormally smooth cortex or irregular cortical infolding, findings that can be overlooked, particularly before the 3rd trimester [21]. This was illustrated in a case involving a fetus with a P/LP variant in *PIK3CA*, where polymicrogyria was not detected at 24 weeks of gestational age, and only suspected at 32 weeks gestational age, subsequently confirmed postnatally [22], highlighting the importance of serial imaging and careful interpretation of subtle findings in early gestation, particularly when genetic testing identifies P/LP variants in *PIK3CA-AKT-mTOR* pathway.

Enlarged extra-axial spaces were observed in two cases with RASopathies, including Noonan syndrome, which limits the ability to draw firm prenatal associations. However, this imaging finding is consistent with postnatal studies of children with RASopathies, where white matter abnormalities, enlargement of extra-axial spaces, and simplified cortical gyral patterns are frequently reported [9]. This potential imaging marker (enlargement of extra-axial spaces) warrants further investigation in larger prenatal cohorts. While this finding is not pathognomonic, its presence should prompt further evaluation, especially when it coexists with systemic features such as cystic hygroma, hydrops, congenital heart disease, polyhydramnios, or renal anomalies. Recent multicenter analyses show that combining these features improves diagnostic rates for RASopathies and supports targeted prenatal genetic testing [10].

Despite the insights gained, this review highlights several limitations. The existing literature on fetal MRI in syndromic megalencephaly consists primarily of case reports and small case series, with only one narrative review. Although the inclusion of a narrative review may raise concerns, it yielded complete, extractable data, and reanalysis without it showed no change in statistical significance. Due to the nature of this systematic review, a standardized interpretation of MRI findings was not feasible; we relied solely on the data and descriptions provided in the original studies. In addition, biometric measurements were not uniformly reported across studies, and in a small number of cases, eligibility was based on the authors’ descriptions of macrocephaly or megalencephaly rather than on independently verifiable head circumference measurements.

This study is subject to referral bias. On one hand, fetuses with isolated macrocephaly may not be referred for fetal MRI, potentially excluding milder cases. On the other hand, fetuses with very severe brain or extracranial anomalies may have undergone TOP based on ultrasound findings alone, leading to their underrepresentation in this MRI cohort. Consequently, the study population may predominantly reflect milder prenatal phenotypes for which MRI was clinically indicated.

The associations between specific imaging features and genetic pathways are based on small case series and should be interpreted as preliminary. The *P*-values reflect limited sample power. Thus, these findings should be considered hypothesis-generating rather than definitive, and require confirmation and validation in larger, prospective cohorts. Future multicenter, international registries and collaborative prospective studies will be essential for validating these genotype-phenotype correlations and establishing their diagnostic utility and standardized imaging protocols.

In many of these reports, fetal MRIs were performed at the end of the second trimester and beginning of the third, which may not fully capture the range of brain anomalies that develop later in gestation, leading to potential underdiagnosis. This limitation is partly due to legal restrictions that limit the gestational age at which pregnancy termination is permitted. It is therefore essential to accurately characterize brain abnormalities at specific gestational stages, particularly in later gestation, while also identifying early indicators of their onset. The implementation of fetal MRI in these cases, due to its accessibility and rapid results, has the potential to significantly improve the accuracy of prenatal diagnoses associated with syndromic megalencephaly. This advancement may contribute to more informed prenatal counseling, enhancing the decision-making process for prospective parents.

Additionally, in many studies, fetal brain imaging was not the primary focus, and MRI assessments lacked sufficient detail. Variability in radiologic involvement and expertise further limits the consistency and reliability of reported findings. The diagnostic value of fetal MRI is highly dependent on the interpreting radiologist’s skill and experience. The lack of standardized protocols across studies underscores the need for uniform and expert-led assessments in future research.

## Conclusion

Fetal MRI is a valuable tool for detecting brain abnormalities in syndromic megalencephaly. Although many findings are nonspecific, certain patterns, such as malformations of cortical development in *PIK3CA*-related overgrowth syndrome and enlargement of the extra-axial spaces in RASopathies, correlate with specific genetic pathways and can guide targeted prenatal diagnosis. Recognition of these imaging features, interpreted alongside ultrasound findings of extracranial anomalies and pregnancy-related factors, and systematic genetic evaluation, may enhance early diagnosis and counseling. Integrating these biomarkers into fetal MRI protocols and adopting a multidisciplinary approach that involves sonography, radiology, genetics, fetal medicine, and neonatology can provide accurate counseling and guide postnatal care. Further research is needed to standardize imaging protocols and clarify genotype–phenotype correlations. Large-scale, prospective studies will refine diagnostic pathways and enhance our ability to provide informed and optimized prenatal care.

## Supplementary information

Below is the link to the electronic supplementary material.

ESM 1 (DOCX 14.5 KB)

ESM 2 (DOCX 27.3 KB)

ESM 3 (DOCX 24.3 KB)

## Data Availability

The data supporting the findings of this study were extracted from previously published studies identified through systematic searches of PubMed and Embase. All data used in the analysis are derived from publicly available sources cited in the reference list. The extracted dataset generated during the current study is available from the corresponding author upon reasonable request.
