# Supplementary material for: Fetal magnetic resonance imaging features of syndromic megalencephaly: a systematic review of imaging-genotype associations
Source: Pediatr Radiol. 2026 Aug 18;56(12):2678–93. doi: 10.1007/s00247-026-06747-3 (PMC13759698; doi:10.1007/s00247-026-06747-3)
Supplement: Supplementary file 1 [file 247_2026_6747_MOESM1_ESM.docx]

**SUPPLEMENTARY MATERIAL**

*Appendix 1 - Search strategy*

PubMed search

Search: **((((macrocephaly AND ((humans[Filter]) AND (2000/1/1:2025/4/30[pdat]) AND (english[Filter]))) AND (fetal or prenatal AND ((humans[Filter]) AND (2000/1/1:2025/4/30[pdat]) AND (english[Filter])))) AND (mri AND ((humans[Filter]) AND (2000/1/1:2025/4/30[pdat]) AND (english[Filter])))) AND (genetic AND ((humans[Filter]) AND (2000/1/1:2025/4/30[pdat]) AND (english[Filter]))) AND ((humans[Filter]) AND (2000/1/1:2025/4/30[pdat]) AND (english[Filter]))) NOT (leukodystrophy or leukoencephalopaty or metabolic AND ((humans[Filter]) AND (2000/1/1:2025/4/30[pdat]) AND (english[Filter])))** Filters: **English, Humans, from 2000/1/1 - 2025/4/30** Sort by: **Publication Date**

((("megalencephaly"[MeSH Terms] OR "megalencephaly"[All Fields] OR "macrocephaly"[All Fields]) AND ("humans"[MeSH Terms] AND 2000/01/01:2025/04/30[Date - Publication] AND "english"[Language]) AND (("fetale"[All Fields] OR "fetally"[All Fields] OR "fetals"[All Fields] OR "fetus"[MeSH Terms] OR "fetus"[All Fields] OR "fetal"[All Fields] OR "foetal"[All Fields] OR ("prenatal"[All Fields] OR "prenatally"[All Fields] OR "prenatals"[All Fields])) AND ("humans"[MeSH Terms] AND 2000/01/01:2025/04/30[Date - Publication] AND "english"[Language])) AND (("magnetic resonance imaging"[MeSH Terms] OR ("magnetic"[All Fields] AND "resonance"[All Fields] AND "imaging"[All Fields]) OR "magnetic resonance imaging"[All Fields] OR "mri"[All Fields]) AND ("humans"[MeSH Terms] AND 2000/01/01:2025/04/30[Date - Publication] AND "english"[Language])) AND (("genetic"[All Fields] OR "genetical"[All Fields] OR "genetically"[All Fields] OR "genetics"[MeSH Subheading] OR "genetics"[All Fields] OR "genetics"[MeSH Terms]) AND ("humans"[MeSH Terms] AND 2000/01/01:2025/04/30[Date - Publication] AND "english"[Language])) AND ("humans"[MeSH Terms] AND 2000/01/01:2025/04/30[Date - Publication] AND "english"[Language])) NOT (("leucodystrophies"[All Fields] OR "leucodystrophy"[All Fields] OR "leukodystrophies"[All Fields] OR "leukodystrophy"[All Fields] OR "leukoencephalopathy"[All Fields] OR ("metabolic"[All Fields] OR "metabolical"[All Fields] OR "metabolically"[All Fields] OR "metabolics"[All Fields] OR "metabolism"[MeSH Terms] OR "metabolism"[All Fields] OR "metabolisms"[All Fields] OR "metabolism"[MeSH Subheading] OR "metabolities"[All Fields] OR "metabolization"[All Fields] OR "metabolize"[All Fields] OR "metabolized"[All Fields] OR "metabolizer"[All Fields] OR "metabolizers"[All Fields] OR "metabolizes"[All Fields] OR "metabolizing"[All Fields])) AND ("humans"[MeSH Terms] AND 2000/01/01:2025/04/30[Date - Publication] AND "english"[Language]))) AND ((humans[Filter]) AND (2000/1/1:2025/4/30[pdat]) AND (english[Filter]))

Embase Search

('macrocephaly'/exp OR 'macrocephaly' OR 'megalencephaly'/exp OR 'megalencephaly') AND 'nuclear magnetic resonance imaging' AND ('fetus disease' OR 'prenatal') AND ('genetic' OR 'overgrowth') AND 'brain' NOT ('leukodystrophy' OR 'leukoencephalopathy' OR 'metabolism' OR 'hydrocephalus') NOT 'conference abstract ':it AND English: la
