# Supplementary material for: Fetal magnetic resonance imaging features of syndromic megalencephaly: a systematic review of imaging-genotype associations
Source: Pediatr Radiol. 2026 Aug 18;56(12):2678–93. doi: 10.1007/s00247-026-06747-3 (PMC13759698; doi:10.1007/s00247-026-06747-3)
Supplement: Supplementary file 2 [file 247_2026_6747_MOESM2_ESM.docx]

*Table S1****:*** *Studies excluded at full text review and reasons for exclusion*

|  | Author Names | Title | Publication Year | Reason for exclusion |
| --- | --- | --- | --- | --- |
| 1 | Zhu S., Zhu H., Liu X., Liu J., Pi G., Yang L., Luo Z., Fan J., Xiong F., Zhang W., Zhou J., Zeng L., Chen A. | Genotype-phenotype correlation of ODLURO syndrome comorbid epilepsy associated with KMT2E variations: Report on a novel case and systematic literature review | 2025 | Did not include a case of a fetus |
| 2 | Ramm E., Perdriolle-Galet E., Bach-Segura P., Bassnagel P., Morel O., Dap M. | Additional value of fetal magnetic resonance imaging for prenatal diagnosis: A retrospective study | 2025 | Did not include genetics for the fetus underwent MRI |
| 3 | Kunpalin Y., Miller E., Raghuram K., Shannon P., Fisher Y., Chau V., Toi A., Chitayat D., Blaser S., Shinar S. | Associations and outcomes of prenatally detected rhombencephalosynapsis | 2024 | Only 2 fetuses with genetic variants, no desciption of their HC. |
| 4 | Bonati M.T., Baldoli C., Taurino J., Marchetti D., Larizza L., Finelli P., Iascone M. | A Novel KIDINS220 Pathogenic Variant Associated with the Syndromic Spastic Paraplegia SINO: An Expansion of the Brain Malformation Spectrum and a Literature Review | 2024 | Did not include a case of a fetus |
| 5 | Asta L., Ricciardello A., Cucinotta F., Turriziani L., Boncoddo M., Bellomo F., Angelini J., Gnazzo M., Scandolo G., Pisanò G., Pelagatti F., Chehbani F., Camia M., Persico A.M. | Clinical, developmental and serotonemia phenotyping of a sample of 70 Italian patients with Phelan-McDermid Syndrome | 2024 | Did not include a case of a fetus |
| 6 | Rodríguez M.A., Echevarría M., Perdomo L., Gómez-Chiari M., García S., Prats P., Serra B., Albaiges G. | Prevalence of corpus callosum pathology in an unselected population. Should assessment of the corpus callosum be included in the routine 20 weeks scan? | 2024 | Did not include cases with macrocephaly with MRI |
| 7 | Tzialla C., Arossa A., Mannarino S., Orcesi S., Veggiotti P., Fiandrino G., Zuffardi O., Errichiello E. | SCN2A and arrhythmia: A potential correlation? A case report and literature review | 2022 | Did not include genetics related to macrocephaly |
| 8 | Serra G., Antona V., Di Pace M.R., Giuffrè M., Morgante G., Piro E., Pirrello R., Salerno S., Schierz I.A.M., Verde V., Corsello G. | Intestinal malrotation in a female newborn affected by Osteopathia Striata with Cranial Sclerosis due to a de novo heterozygous nonsense mutation of the AMER1 gene | 2022 | Did not include a case of a fetus |
| 9 | Goergen S.K., Fahey M.C. | Prenatal MR Imaging Phenotype of Fetuses with Tuberous Sclerosis: An Institutional Case Series and Literature Review | 2022 | Did not include confirmation of genetic variant |
| 10 | Kobets A., Lee R.P., Oriko D., Jackson E., Robinson S., Cohen A., Groves M.L. | Dual Myelomeningoceles in Twins: Case Report, Review, and Insights for Etiology | 2021 | Macrocephaly secondary to hydrocephalus, no confirmation of genetic variant |
| 11 | Lerman-Sagie T., Pogledic I., Leibovitz Z., Malinger G. | A practical approach to prenatal diagnosis of malformations of cortical development | 2021 | Did not include genetics related to macrocephaly, nor macrocephalic cases described |
| 12 | Santirocco M., Plaja A., Rodó C., Valenzuela I., Arévalo S., Castells N., Abuli A., Tizzano E., Maiz N., Carreras E. | Chromosomal microarray analysis in fetuses with central nervous system anomalies: An 8-year long observational study from a tertiary care university hospital | 2021 | Did not include an MRI description and macrocephaly for the cases |
| 13 | Ho M.-L., Mansukhani S.A., Brodsky M.C. | Prenatal or Perinatal Injury? Diagnosing the Cortically Blind Infant | 2020 | Did not include a case of macrocephaly or fetus |
| 14 | Thiffault I., Atherton A., Heese B.A., Abdelmoity A.T., Pawar K., Farrow E., Zellmer L., Miller N., Soden S., Saunders C. | Pathogenic variants in KPTN gene identified by clinical whole-genome sequencing | 2020 | Did not include a case of a fetus |
| 15 | Vera G., Sorlin A., Delplancq G., Lecoquierre F., Brasseur-Daudruy M., Petit F., Smol T., Ziegler A., Bonneau D., Colin E., Mercier S., Cogné B., Bézieau S., Edery P., Lesca G., Chatron N., Sabatier I., Duban-Bedu B., Colson C., Piton A., Durand B., Capri Y., Perrin L., Wiesener A., Zweier C., Maroofian R., Carroll C.J., Galehdari H., Mazaheri N., Callewaert B., Giulianno F., Zaafrane-Khachnaoui K., Buchert-Lo R., Haack T., Magg J., Rieß A., Blandfort M., Waldmüller S., Horber V., Leonardi E., Polli R., Turolla L., Murgia A., Frebourg T., Lebre A.S., Nicolas G., Saugier-Veber P., Guerrot A.-M. | Clinical and molecular description of 19 patients with GATAD2B-Associated Neurodevelopmental Disorder (GAND) | 2020 | Did not include a case of a fetus |
| 16 | Baron J., Mastrolia S.A., Shelef I., Tirosh D., Mijalovsky A., Ben-Harush Y., Hershkovitz R. | Fetal wide subarachnoid space and its outcome in cases of macrocephaly without ventriculomegaly | 2020 | Did not include an MRI description |
| 17 | Huo L., Teng Z., Wang H., Liu X. | A novel splice site mutation in AP1S2 gene for X-linked mental retardation in a Chinese pedigree and literature review | 2019 | Did not include a case of a fetus |
| 18 | Griffiths P.D., Bradburn M., Mandefield L., Mooney C., Jarvis D. | The rate of brain abnormalities on in utero MRI studies in fetuses with normal ultrasound examinations of the brain and calculation of indicators of diagnostic performance | 2019 | Did not include genetics |
| 19 | Allali S., Dorboz I., Samaan S., Slama A., Rambaud C., Boespflug-Tanguy O., Sarret C. | Mutation in the AGK gene in two siblings with unusual Sengers syndrome | 2017 | Did not include a case of a fetus |
| 20 | Nagaraj U.D., Hopkin R., Schapiro M., Kline-Fath B. | Prenatal and postnatal evaluation of polymicrogyria with band heterotopia | 2017 | Did not include genetics |
| 21 | Gowda V.K., Bhat M.D., Srinivasan V.M., Prasad C., Benakappa A., Faruq M. | A case of Canavan disease with microcephaly | 2016 | Macrocelpahly ralated to leukodystropy |
| 22 | Lerman-Sagie T., Leibovitz Z. | Malformations of Cortical Development: From Postnatal to Fetal Imaging | 2016 | Did not include genetics for the fetus underwent MRI |
| 23 | Frassoni C., Avagliano L., Inverardi F., Spaccini L., Parazzini C., Rustico M.A., Bulfamante G., Righini A. | Familial Precocious Fetal Abnormal Cortical Sulcation | 2016 | Did not include a case of macrocephaly |
| 24 | Stutterd C.A., Leventer R.J. | Polymicrogyria: A common and heterogeneous malformation of cortical development | 2014 | Did not include a case of a fetus |
| 25 | Pirzadeh Z. | Glutaric acidemia, a preliminary survey on iranian children | 2012 | Macrocelpahly ralated to Metabolic disease |
| 26 | Papetti L., Tarani L., Nicita F., Ruggieri M., Mattiucci C., Mancini F., Ursitti F., Spalice A. | Macrocephaly-capillary malformation syndrome: Description of a case and review of clinical diagnostic criteria | 2012 | Did not include a case of a fetus |
| 27 | Righini A., Parazzini C., Doneda C., Avagliano L., Arrigoni F., Rustico M., Consonni D., Re T.J., Bulfamante G., Triulzi F. | Early formative stage of human focal cortical gyration anomalies: Fetal MRI | 2012 | Did not include genetics |
| 28 | Kraker J.B., Abdel-Hamid H., Lacomis D. | Neuromuscular pathology unknown | 2010 | Macrocelpahly ralated to leukodystropy |
| 29 | Parazzini C., Righini A., Lalatta F., Bianchini E., Triulzi F. | Frontal bilateral megalencephaly: Fetal and autopsy MR evaluation of an unclassified malformation | 2005 | Did not include genetics |
| 30 | Akcar N., Adapinar B., Dinleyici C., Durak B., Özkan I.R. | A case of macrocephaly-cutis marmorata telangiectatica congenita and review of neuroradiologic features | 2004 | Did not include a case of a fetus |
| 31 | Balci S., Tekşam Ö., Gedik Ş. | Megalocornea, macrocephaly, mental and motor retardation: MMMM syndrome (Neuhäuser syndrome) in two sisters with hypoplastic corpus callosum | 2002 | Did not include an MRI description |
| 32 | Chen C.-P., Lin S.-P., Chang T.-Y., Chiu N.-C., Shih S.-L., Lin C.-J., Wang W., Hsu H.-C. | Perinatal imaging findings of inherited Sotos syndrome | 2002 | Did not include a fetal MRI description |
| 33 | Abdel-Salam GMH, Esmail A, Nagy D, Abdel-Ghafar SF, Abdel-Hamid MS. | Novel homozygous ESAM variants in two families with perinatal strokes showing variable neuroradiologic and clinical findings | 2025 | Did not include a case of a fetus |
| 34 | Vallin AL, Grévent D, Bessières B, Salomon LJ, Legeai-Mallet L, Cormier-Daire V, Baujat G, Ville Y, Faure-Bardon V. | Foetal achondroplasia: Prenatal diagnosis, outcome and perspectives | 2025 | Did not include confirmation of genetic variant for the born children with macrocephaly nor a specific description of their fetal MRI |
| 35 | Su XR, Ma B, Zhang C, Li TG, Han BL, Wu WR, Nie F. | Prenatal Ultrasound Diagnosis of Megalencephaly-Polymicrogyria-Polydactyly-Hydrocephalus Syndrome with Persistent Hyperplastic Primary Vitreous: A Case Report | 2024 | Did not include a case of fetal MRI |
| 36 | Rinaldi B, Cesaretti C, Boito S, Villa R, Guerneri S, Borzani I, Rizzuti T, Marchetti D, Conte G, Cinnante C, Triulzi F, Persico N, Iascone M, Natacci F. | Family history is key to the interpretation of exome sequencing in the prenatal | 2022 | Did not include MRI for the genetic case |
| 37 | Muhsin E, Basak G, Banu D, Alper G, Mustafa S. | Neurodevelopment and Genetic Evaluation of Sotos Syndrome Cases with a Novel Mutation: a Single-Center Experience | 2022 | Did not include a case of a fetus |
| 38 | Traub ES, Sheppard SE, Dori Y, Burns KD, Zackai EH, Ware SM, Landis BJ, Li D, Weaver DD. | Chromosome 4q28.3q32.3 duplication in a patient with lymphatic malformations, craniosynostosis, and dysmorphic features | 2021 | Did not include a case of a fetus |
| 39 | Nakamura Y, Togawa Y, Okuno Y, Muramatsu H, Nakabayashi K, Kuroki Y, Ieda D, Hori I, Negishi Y, Togawa T, Hattori A, Kojima S, Saitoh S. | Biallelic mutations in SZT2 cause a discernible clinical entity with epilepsy, developmental delay, macrocephaly and a dysmorphic corpus callosum | 2018 | Did not include a case of a fetus |
| 40 | Karaer K, Rosti RO, Torun D, Sanal HT, Guran S. | Macrocephaly with multiple epiphyseal dysplasia: a second example of Al Gazali-Bakalinova syndrome? | 2012 | Did not include a case of a fetus |
| 41 | Di Donato N, Riess A, Hackmann K, Rump A, Huebner A, von der Hagen M, Hahn G, Schrock E, Tinschert S. | Macrocephaly, obesity, mental (intellectual) disability, and ocular abnormalities: alternative definition and further delineation of MOMO syndrome | 2012 | Did not include a case of a fetus |
| 42 | Ali BR, Silhavy JL, Akawi NA, Gleeson JG, Al-Gazali L. | A mutation in KIF7 is responsible for the autosomal recessive syndrome of macrocephaly, multiple epiphyseal dysplasia and distinctive facial appearance | 2012 | Did not include a cases of macrocephaly |
| 43 | Khattab M, Xu F, Li P, Bhandari V. | A de novo 3.54 Mb deletion of 17q22-q23.1 associated with hydrocephalus: a case report and review of literature | 2011 | Secondary macrocephaly (hydrocephalus) |
| 44 | Su PH, Yu JS, Chen SJ, Chen JY, Tsao TF. | Persistent falcine sinus and unilateral renal agenesis in a girl with Sotos syndrome | 2011 | Did not include a case of a fetus |
| 45 | Kasnauskiene J, Cimbalistiene L, Ciuladaite Z, Preiksaitiene E, Kučinskienė ZA, Hettinger JA, Sismani C, Patsalis PC, Kučinskas V. | De novo 5q35.5 duplication with clinical presentation of Sotos syndrome | 2011 | Did not include a case of a fetus |
| 46 | Isaacs H Jr. | Perinatal neurofibromatosis: two case reports and review of the literature | 2010 | Did not include a case of a fetus |
| 47 | Gripp KW, Hopkins E, Vinkler C, Lev D, Malinger G, Lerman-Sagie T, Dobyns WB. | Significant overlap and possible identity of macrocephaly capillary malformation and megalencephaly polymicrogyria-polydactyly hydrocephalus syndromes | 2009 | Did not include genetics |
| 48 | Scelsa B, Bedeschi FM, Guerneri S, Lalatta F, Introvini P. | Partial trisomy of 7q: case report and literature review | 2008 | Did not include a case of a fetus |
| 49 | Tohyama J, Akasaka N, Saito N, Yoshimura J, Nishiyama K, Kato M. | Megalencephaly and polymicrogyria with polydactyly syndrome | 2007 | Did not include a case of a fetus, no genetics |
| 50 | Bruner JP, Davis G, Tulipan N. | Intrauterine shunt for obstructive hydrocephalus--still not ready | 2006 | Secondary macrocephaly (hydrocephalus) |
| 51 | Battaglia A, Chines C, Carey JC. | The FG syndrome: report of a large Italian series | 2006 | Did not include a case of a fetus |
| 52 | Nokelainen P, Heiskala H, Raininko R, Autti T, Wirtavuori K, Häkkinen AM, Flint J. | Two brothers with macrocephaly, progressive cerebral atrophy and abnormal white matter, severe mental retardation, and Lennox-Gastaut spectrum type epilepsy: an inherited encephalopathy of childhood? | 2001 | Macrocelpahly ralated to leukodystropy |
| 53 | Malinger G, Lev D, Ben-Sira L, Hoffmann C, Herrera M, Viñals F, Vinkler H, Ginath S, Biran-Gol Y, Kidron D, Lerman-Sagie T | Can syndromic macrocephaly be diagnosed in utero? | 2010 | Did not include genetics |
| 54 | Brady LI, DeFrance B, Tarnopolsky M | Pre- and Postnatal Characterization of Autosomal Recessive KIDINS220-Associated Ventriculomegaly | 2022 | Secondary macrocephaly (hydrocephalus) |
