# Supplementary material for: Fetal magnetic resonance imaging features of syndromic megalencephaly: a systematic review of imaging-genotype associations
Source: Pediatr Radiol. 2026 Aug 18;56(12):2678–93. doi: 10.1007/s00247-026-06747-3 (PMC13759698; doi:10.1007/s00247-026-06747-3)
Supplement: Supplementary file 3 [file 247_2026_6747_MOESM3_ESM.docx]

Table S2: Risk of bias assessment using the Joanna Briggs Institute checklists

| Case studies |  |  |  |  |  |  |  |  |  |  |
| --- | --- | --- | --- | --- | --- | --- | --- | --- | --- | --- |
| **Authors** | **Year** | 1. Demographic clearly described | 2. History clearly described presented as a timeline | 3. Clinical condition at presentation clearly described | 4. Diagnostic tests and the results clearly described | 5. Intervention or treatment procedure clearly described | 6. Post-intervention clinical condition clearly described* | 7. Adverse events identified and described | 8. Takeaway lessons | Score |
| Abell et al. | 2020 | Yes | Yes | Yes | Yes | Yes | Yes | Yes | Yes | 8/8 |
| Andrade et al. | 2025 | Yes | Yes | Yes | Yes | No | Yes | Yes | Yes | 7/8 |
| Beltrami et al. | 2022 | Yes | Yes | Yes | Yes | No | Yes | Yes | Yes | 7/8 |
| Chen et al. | 2024 | Yes | Yes | Yes | Yes | Yes | No | Yes | Yes | 7/8 |
| Del Campos Braojos & Guimaraes | 2018 | Yes | Yes | Yes | Yes | Yes | Yes | Yes | Yes | 8/8 |
| Giovannangeli et al. | 2024 | Yes | Yes | Yes | Yes | Yes | No | Yes | Yes | 7/8 |
| Hague et al. | 2017 | Yes | Yes | Yes | Yes | Yes | Yes | Yes | Yes | 8/8 |
| Lei et al. | 2023 | Yes | Yes | Yes | Yes | Yes | No | Yes | Yes | 7/8 |
| Liu et al. | 2023 | Yes | Yes | Yes | Yes | Yes | No | Yes | Yes | 7/8 |
| Malinger et al. | 2021 | Yes | Yes | Yes | Yes | Yes | No | Yes | Yes | 7/8 |
| Mori et al. | 2022 | Yes | Yes | Yes | Yes | Yes | Yes | Yes | Yes | 8/8 |
| Porwal et al. | 2022 | Yes | Yes | Yes | Yes | Yes | Yes | Yes | Yes | 7/8 |
| Quinlan-Jones et al. | 2017 | Yes | Yes | Yes | Yes | Yes | Yes | Yes | Yes | 8/8 |
| Shear et al. | 2024 | Yes | Yes | Yes | Yes | No | Unclear | Yes | Yes | 6/8 |
| Speksnijder et al. | 2013 | Yes | Yes | Yes | Yes | Yes | Yes | Yes | Yes | 8/8 |
| Swarr et al. | 2013 | Yes | Yes | Yes | Yes | Yes | Yes | Yes | Yes | 8/8 |
| Valduga et al. | 2015 | Yes | Yes | Yes | Yes | Yes | Yes | Yes | Yes | 8/8 |

**Case series**

| **Authors** | **Year** | 1. Clear criteria for inclusion | 2. Condition measured in a standard way | 3. Valid methods used for identification of the condition | 4. Consecutive inclusion of participants | 5. Complete inclusion of participants | 6. Clear reporting of the demographics | 7. Clear reporting of clinical information | 8. Outcomes or follow up results clearly reported | 9. Clear reporting of presenting site demographic information | 10. Statistical analysis appropriate? | Score |
| --- | --- | --- | --- | --- | --- | --- | --- | --- | --- | --- | --- | --- |
| Bourgon et al. | 2022 | Yes | Yes | Yes | Yes | Yes | Yes | Yes | Yes | Yes | No | 9/10 |
| Di Stasi et al. | 2021 | Yes | Yes | Yes | Unclear | Yes | Yes | Yes | Yes | Yes | No | 8/10 |
| Goergen et al. | 2021 | Yes | Yes | Yes | Yes | Yes | Yes | Yes | Yes | Yes | No | 9/10 |
| Neri et al. | 2022 | Yes | Yes | Yes | Unclear | Yes | Yes | Yes | Yes | No | No | 7/10 |
| Rodan et al. | 2018 | Yes | Yes | Yes | Unclear | Yes | Yes | Yes | Yes | No | No | 7/10 |
| Saliba et al. | 2018 | Yes | Yes | Yes | Unclear | Yes | Yes | Yes | Yes | Unclear | No | 7/10 |
| Yu et al. | 2025 | Yes | Yes | Yes | Yes | Yes | Yes | Yes | Yes | No | No | 7/10 |

Cohort studies

| **Authors** | **Year** | 1. Two groups similar recruited from the same population | 2. Exposures measured similarly to assign people to both exposed | 3. Exposure measured in a valid way | 4. Confounding factors identified | 5. Strategies to deal with confounding factors stated | 6. Participants free of the outcome at the start of the study | 7. Outcomes measured in a valid way | 8. Follow up time reported and sufficient to be long enough for outcomes to occur | 9. Follow up complete/ reasons to loss to follow up described | 10. Strategies to address incomplete follow up utilized | 11. Appropriate statistical analysis | Score |
| --- | --- | --- | --- | --- | --- | --- | --- | --- | --- | --- | --- | --- | --- |
| Libzon et al. | 2024 | Yes | Yes | Yes | Yes | Yes | Yes | Yes | Yes | Yes | Yes | Yes | 11/11 |

Narrative Review

| **Authors** | **Year** | 1. source of the opinion clearly identified | 2. source of opinion have standing in the field of expertise | 3 Interests of the relevant population the central focus of the opinion | 4. Stated position the result of an analytical process, logic in the opinion expressed? | 5. reference to the extant literature | 6. incongruence with the literature/sources logically defended |  |
| --- | --- | --- | --- | --- | --- | --- | --- | --- |
| Papaioannou & Garel | 2023 | Yes | Yes | Yes | Yes | Yes | Not applicable | 5/6 |

*” Post-intervention” was considered “yes” when there was a description of the child’s development or postnatal MR

**Appendix 2 – Clarification on Use of Joanna Briggs Institute Checklists**

For case reports and case series, the following adaptations were applied when using the Joanna Briggs Institute (JBI) critical appraisal tools:

Question 5 – Intervention Described

An intervention was considered present if it was actively performed on the fetus or directly related to fetal care, such as fetal sampling procedures (e.g., amniocentesis, cordocentesis). therapeutic fetal interventions, termination of pregnancy, preterm delivery due to fetal indication/

Fetal imaging was not classified as an intervention but rather as a diagnostic assessment.

Question 6 – Post-Intervention Clinical Condition Reported

Follow-up of the fetal condition was deemed present when the study included post-intervention outcomes, such as postnatal MRI findings, postmortem autopsy results, and clinical outcome at delivery.
